# Noncommercial US Funders’ Policies on Trial Registration, Access to Summary Results, and Individual Patient Data Availability

**DOI:** 10.1001/jamanetworkopen.2018.7498

**Published:** 2019-01-25

**Authors:** Evelyn P. Whitlock, Kelly M. Dunham, Kimberly DiGioia, Emily Lazowick, Theresa C. Gleason, David Atkins

**Affiliations:** 1 Patient-Centered Outcomes Research Institute, Washington, DC; 2 Office of Research and Development, Clinical Science Research & Development Service, US Department of Veterans Affairs, Washington, DC; 3 Office of Research and Development, Health Services Research & Development Service, US Department of Veterans Affairs, Washington, DC

## Abstract

**Importance:**

Incomplete information about existing research is an underlying cause of research waste. National and international initiatives and requirements have been launched to address this issue.

**Objectives:**

To characterize current clinical trial transparency policies among the largest noncommercial US funders and examine whether the policies are concordant with international funders.

**Design, Setting, and Participants:**

This retrospective review of public information used methods developed for documenting funder policies internationally; 2 researchers searched each funder’s website and Google between May and November 2018 to locate trial transparency policies for 10 top US funders. Key informants at each funding organization were contacted by email and given 3 or more weeks to review and confirm or correct the findings. Nonresponders were contacted 2 or more additional times. Descriptive statistics were calculated to summarize the findings. The study was conducted using publicly available policy information with findings confirmed by funder representatives where possible. Participants included top 10 noncommercial US health research funders with the highest reported investment in health research (2013 dollars) who fund clinical trials. Data analysis was conducted from November 6, 2018, to November 23, 2018.

**Exposures:**

Availability of policies addressing each of the 3 key trial transparency domains as specified by the World Health Organization in 2017.

**Main Outcomes and Measures:**

Independent assessment by 2 investigators of availability (yes or no) of a policy addressing registration for trials, sharing of summary results, and individual participant data sharing activities; requirements (yes, no, or supportive statement) of these policies in terms of completeness, timeliness, public access, and provision of additional technical or financial support to meet data sharing requirements; description (yes or no) of internal monitoring for policy adherence.

**Results:**

All 10 funders acknowledged the outreach. One funder who indicated that less than 1% of their research funding goes to clinical trials was removed. Six (67%) of the remaining 9 top US funders have a publicly available written policy for all 3 major trial transparency domains. The most comprehensive trial transparency practice among US funders addresses summary results sharing as follows: 8 of 9 US funders (89%) have a policy, 5 of 9 US funders (56%) require reporting of summary results within 1 year, and 6 of 9 US funders (67%) monitor compliance with their summary results sharing policy. For clinical trial registration, 7 of 9 US funders (78%) have a policy and 5 of 9 US funders (56%) require registration and monitor trial registration to measure adherence to the policy.

**Conclusions and Relevance:**

In this study, overall the proportion of US funders with policies and practices to support trial transparency in this sample was similar or compared favorably with the larger international sample of noncommercial funders recently reported.

## Key Points

**Question:**

What are the current policies for clinical trial registration, summary results sharing, and individual patient data sharing among the top 10 noncommercial US health research funders?

**Findings:**

In this review study, 6 of 9 (67%) of the top US funders have a publicly available written policy addressing all 3 major trial transparency domains. However, fewer US funders require specific transparency actions in these domains (11%-56%) or monitor compliance with their policies (56%-67%).

**Meaning:**

More work remains to be done to ensure timely implementation and enforcement of clinical trial transparency initiatives to reduce waste and realize public value from clinical research investments.

## Teaser

This review study examines noncommercial US funders’ policies on clinical trial transparency, including clinical trial registration, summary results sharing, and individual patient data sharing activities.

## Introduction

Over the past 20 years,^1^ national and international initiatives have been launched to improve clinical trial registration and reporting.^2,3,4^ Incomplete information about existing research is an underlying cause of research waste^5^ and undermines credible, efficient production of research to support health care decision making.^6^ Trial registries afford the means to capture a minimum, structured information set from all ongoing and completed clinical trials in humans and to make these publicly accessible. Efforts in the United States have been greatly enhanced by the creation of ClinicalTrials.gov in 2000 by the National Institutes of Health (NIH), which expanded to incorporate results reporting in 2008; ClinicalTrials.gov currently houses approximately a quarter of a million trial records, representing about two-thirds of clinical trials registered globally.^7^

Under Title VIII of the US Food and Drug Administration Amendments Act of 2007 (FDAAA)^8^ and subsequent rule making by the US Department of Health and Human Services in 2016,^9^ US research sponsors and principal investigators have a clear, legally binding trial reporting obligation. They must register and report summary results from applicable clinical trials of FDA-regulated drug, biologic, and device products on ClinicalTrials.gov within set timelines, with possible penalties to be imposed for failure to meet these requirements.^10^ Nonetheless, despite growing requirements and opportunities for trial registry and reporting, US academic institutions (a common research sponsor in the United States) may be incompletely prepared^11^ or lag in their duty to completely report their clinical trials’ planning, conduct, and results to the public in timely and transparent ways.^7^

It should be noted that the FDAAA regulatory requirements for reporting specific clinical trials in the United States directly apply to research sponsors or their delegates and not to funding agencies. However, possible penalties allowed under the law include withholding of research funding by the US Department of Health and Human Services agencies. And, as the largest funder of clinical trials (the NIH) has demonstrated, federal and nonfederal funders can play a key role in ensuring good research practices for trial registration and reporting; the NIH voluntarily expanded registration and reporting requirements to apply to all NIH-funded clinical trials and not just those required by the FDAAA, with compliance and enforcement provisions.^12^ Alongside regulators, research funders may be best positioned within the health research system to provide both the motivation and the means for achieving more complete clinical trial registration and reporting, an important component of reducing research waste.^13^

To examine the current commitment of health research funders to ensuring clinical trial availability, DeVito and colleagues^14^ recently reported on trial transparency policies for the top 18 noncommercial clinical trials funders globally (by 2013 research expenditures). The authors audited each funder’s policy requirements for 3 key domains of trial transparency and reporting (trial registration, summary results sharing, and individual patient data sharing), as specified in 2017 by the World Health Organization^4^ and others.^7^ In that international sample, 8 funders (44%) had a policy addressing each of the 3 domains, although fewer had specific requirements or audited trialists’ performance. Just 3 of the 18 funders examined were from the United States. Due to the international nature of the sample, not all funders operate under similar regulatory requirements.

To document more completely how US funders are participating, particularly since the FDAAA, we undertook further evaluation of current trial transparency policies among an expanded set of noncommercial US funders using the methods from the international review. Specifically, exploring whether US funders’ public policies addressing trial reporting transparency are generally concordant or appear to deviate from the international community was of interest.

## Methods

Using a public website, we identified 81 health research funding organizations in the United States and 73 of these had annual health research expenditures reported for 2013.^15^ We eliminated 13 additional funders for whom health-related clinical trials are not the norm (eg, the US Department of Labor and the US Department of the Interior) and 1 funder (ie, Howard Hughes Medical Institute) who had previously reported they do not fund clinical trials.^14^ Of the remaining 60 funders, 10 were selected with the highest reported investment in health research for review (Table 1); altogether, this group’s investment in health research exceeded 80% of the total US health research dollars reported for 2013. The listing of the top 10 US clinical trial research funders included the 3 previously examined US funders.^14^ Using the methods developed by DeVito et al,^14^ 2 researchers searched each funder’s website and Google in May and June 2018 and again in October 2018 to locate trial transparency policies for the 10 US funders. Funder policies and extracted structured data were reviewed to assess whether the relevant policy met each of the 11 prespecified criteria as defined by DeVito et al.^14^ Policies were extracted verbatim and discrepancies in coding interpretation were resolved by consensus using a third researcher, as necessary. Discrepancies were infrequent and generally involved the identification of the appropriate, relevant policy passages from multiple documents and not coding interpretation. Key informants at each funding organization were identified and contacted by email after each round of Google searching and policy updating, with at least 3 weeks to review and confirm or correct the study’s findings. Nonresponders were contacted 2 or more additional times. Updated findings based on written policies that diverge from the previous work by DeVito et al^14^ were confirmed by key informants. Descriptive statistics were used to summarize the findings, including those who did not confirm our findings. This study was determined to be exempt from oversight by the Advarra institutional review board as this was a retrospective review of publicly available information.

**Table 1.** Annual Health Research Expenditures of the Top 10 Clinical Trials Funders in the United States

Organization	Annual Health Research Expenditures (2013), US$ in Millions
National Institutes of Health	26 081.3
US Department of Veterans Affairs	582.0
Bill & Melinda Gates Foundation	462.6
Centers for Disease Control and Prevention	440.7
Congressionally Directed Medical Research Program^a^	409.0
Agency for Healthcare Research and Quality	371.3
US Food and Drug Administration	357.9
Patient-Centered Outcomes Research Institute	202.0
American Cancer Society	162.5
American Heart Association	135.6
Total	29 204.9

^a^ Department of Defense health research expenditures total $1426.7 million; policies are represented by the Congressionally Directed Medical Research Program.

## Results

All funders acknowledged our outreach. Six of 9 funders (67%) provided comments or corrections to the study’s findings. One funder who indicated that less than 1% of their research funding goes to clinical trials was removed. Based on the study’s findings (Table 2), 6 of 9 (67%) of the top US funders have a publicly available written policy for all 3 major trial transparency domains; 2 others have policies for 2 of the 3 domains. Despite multiple attempts, policies for any domain for 3 funders could not be confirmed.

**Table 2.** Summary Results of Audit of Trial Transparency Policies of US Noncommercial Funders of Clinical Trials

Funder	Responder to Outreach	Trial Transparency Policies										
		Trial Registration			Summary Results Sharing				Individual Patient Data Sharing			
		Has Policy	Requires Registration	Monitors Compliance	Has Policy	Requires Sharing	Specifies Timeline for Sharing	Monitors Compliance	Has Policy	Requires Sharing	Offers Sharing Support	Monitors Compliance
NIH^a^	Yes	Yes	Yes	Yes	Yes	Yes	1 y	Yes	Yes	Supportive	Yes	Yes
VA	Yes	Yes	Yes	Yes	Yes	Yes	1y	Yes	Yes	Supportive	Yes	No
Gates^a^	Yes	No	No	No	Yes	Supportive	No	No	Yes	Supportive	No	Yes
CDC^b^	Yes	Yes	Supportive	No	Yes	Supportive	1 y	No	Yes	Supportive	Yes	Yes
CDMRP^a^^,^^c^	Yes	Yes	Yes	Yes	Yes	Supportive	1 y	Yes	Yes	Supportive	Yes	Yes
AHRQ^b^	Yes	Yes	Supportive	No	Yes	Yes	1 y	Yes	Yes	Yes	Yes	No
FDA^b^	Yes	Yes	Yes	Yes	Yes	Yes	1 y	Yes	No	No	No	No
PCORI	Yes	Yes	Yes	Yes	Yes	Yes	1 y	Yes	Yes	Supportive	Yes	Yes
ACS	Yes	No	No	No	No	No	No	No	No	No	No	No
Total yes (% yes)	10 (100)	7 (78)	5 (56); Supportive: 2 (22)	5 (56)	8 (89)	5 (56); Supportive: 3 (33)	7 (78)	6 (67)	7 (78)	1 (11); Supportive: 6 (67)	6 (67)	5 (56)

Abbreviations: ACS, American Cancer Society; AHRQ, Agency for Healthcare Research and Quality; CDC, Centers for Disease Control and Prevention; CDMRP, Congressionally Directed Medical Research Program; FDA, US Food and Drug Administration; Gates, Bill & Melinda Gates Foundation; NIH, National Institutes of Health; PCORI, Patient-Centered Outcomes Research Institute; VA, US Department of Veterans Affairs.

^a^ Funder was included in the previous analysis by DeVito et al.^14^

^b^ Funder acknowledged outreach but did not confirm or correct our findings. The CDC and FDA did not list information about trial registration requirements on their website, but the CDC had policies on open access. Coded responses for the CDC reflect the assumptions that some but not most trials are subject to the Food and Drug Administration Amendments Act of 2007. Coded responses for the FDA reflect the Food and Drug Administration Amendments Act of 2007 requirements for applicable clinical trials assumed to apply to most trials. The AHRQ responses reflect publicly available policies from their website.

^c^ The Department of Defense represented by the CDMRP.

For each of the 3 transparency domains, most top US funders have a policy in place; however, these funders less clearly require specific trialist actions, offer support for data sharing, or audit for compliance with their policy. The most comprehensive trial transparency practice among US funders addresses summary results sharing as follows: 8 of 9 US funders (89%) have a policy, 5 of 9 US funders (56%) require reporting of summary results within 1 year, and 6 US funders (67%) monitor compliance with their summary results sharing policy. For clinical trial registration, 7 US funders (78%) have a policy and 5 US funders (56%) require registration and monitor trial registration to measure adherence to the policy. The least developed transparency policy and practice among noncommercial US funders concerns individual patient data sharing. Although 7 US funders (67%) have a policy, only 1 funder (11%) currently requires sharing individual patient data from clinical trials, while the other 6 US funders (67%) make supportive statements in their policies about sharing individual patient data. Six US funders (67%) offer some technical or financial resources to support awardee individual patient data sharing efforts.

Findings for 3 funders evaluated by DeVito et al^14^ were separately confirmed. The study’s findings only differed for Congressionally Directed Medical Research Programs whose central scientific office provided several rounds of communication, policy text (including references to FDAAA and NIH), and reviewed this study’s coding; final coding reflects more affirmative results than DeVito et al^14^ for trial registration requirements and compliance monitoring, timeline for sharing summary results, and monitoring for compliance with individual participant data sharing policies.

## Discussion

Overall, the proportion of funders with policies and practices to support trial transparency in this US sample was similar or compared favorably with the larger international sample of noncommercial funders recently reported.^14^ This is despite the fact that the responsibility for US funders may be somewhat lessened compared with international colleagues by the presence of clear regulatory requirements for sponsors through the FDAAA. To some degree, US funders in addition to the NIH appear to have embraced the opportunity to use their leverage to make trial-related efforts more transparent and accessible in general. Although based on small samples, it appears that a somewhat greater proportion of US funders require investigators to report summary results as compared with results for international funders (56% vs 44%) reported by DeVito et al^14^ and specify a timeline for sharing study results (78% vs 22%), usually within 1 year of publication or primary completion date.^14^ This may reflect the clear expectations for timely reporting, usually within 1 year, specified through the FDAAA. Relatively similar small proportions of US and international funders require individual participant data sharing or offer technical or financial resources to support individual participant data sharing. However, the data sharing aspect of trials transparency is changing rapidly and the policies for 1 funder (Patient-Centered Outcomes Research Institute) were updated during the 4 months between funder contacts.^16^ As the research enterprise accepts the idea that making individual participant data available for further research fulfills an ethical obligation to trial participants,^17^ and is something participants generally support,^18^ scientists can turn their focus to clarifying minimal requirements for ensuring anonymity, appropriate use according to informed consent, and data sharing (eg, including making analytic code and other key resources available). A research culture that readily shares and reuses research data will be better positioned to more fully support the scientific imperatives of replication and reproducibility.^6^

### Limitations

This study had several limitations. The findings may underrepresent some funders’ activities since they are based only on publicly available policies and findings for 3 organizations could not be confirmed, despite repeated efforts. Given existing regulatory requirements, funders may be presumed to have internal policies that reflect the FDAAA at minimum. Similarly, policy availability was the focus of this investigation, not impact or organizational performance; the existence of policies does not necessarily result in the benefits they promise, unless specific requirements are coupled with compliance and enforcement activities. The data represent a snapshot in time and policies in this arena are quite dynamic and challenging to interpret, as shown by small differences between the findings of DeVito et al^14^ and the present study, as well as evolution of some policies during the time of the study’s investigation. The policies of the top 10 noncommercial funders of health research in the United States in 2013 as a proxy for the top noncommercial funders of clinical trials were examined. While funders who clarified they fund no or very limited clinical trials were eliminated, this proxy is nonetheless inexact. This group accounted for a large portion ($29 billion) and proportion but not all US health research expenditures.^15^ Nonetheless, improving the culture of trial transparency may be efficiently accomplished by focusing first on the funders with the largest current impact.

## Conclusions

Biomedical research funders in the United States and elsewhere can make important contributions to improving the transparency of funded clinical trials. Complementing the efforts of regulators, journal editors, and research sponsors, funders can implement policies with specific registration and reporting requirements and monitor their awardees for completion. Funders can consider withholding additional funding from researchers or institutions who fail to perform. A convergence of efforts across the research system is required to shift the culture of research to embrace greater transparency and public accountability. As another tangible benefit, through requiring trial registration and the dissemination of results, funders can increase efficiency by allowing others in the biomedical research ecosystem the information needed to avoid unnecessary duplication and to build on existing research. As key actors in the research ecosystem, research funders are banding together to encourage and support adaptation of responsible research funding practices across many areas, including trials transparency, in furtherance of maximal public good from funded clinical research.^19^
